# Characteristics of violent adolescents examined in a forensic psychiatric assessment

**DOI:** 10.1192/j.eurpsy.2021.612

**Published:** 2021-08-13

**Authors:** D. Jardak, S. Omri, I. Chaari, N. Smaoui, R. Feki, J. Ben Thabet, L. Zouari, N. Charfi, M. Maalej Bouali, M. Maalej

**Affiliations:** 1 Psychiatrie C, Hedi chaker hospital, sfax, Tunisia; 2 Psychiatry C Department, Hedi chaker University hospital, sfax, Tunisia

## Abstract

**Introduction:**

The acts of violence committed by adolescents are becoming increasingly more common, generating problems of a diverse nature.

**Objectives:**

To study the main characteristics of violent adolescent examined in a forensic psychiatric assessment.

**Methods:**

This is a retrospective study which examined the expert files of the subjects aged between 14 and 20 years charged with violence, which were examined in the context of criminal psychiatric expertise in the psychiatry department of Hedi Chaker University Hospital in Sfax (Tunisia), between January 2002 and December 2018.

**Results:**

Our study included 34 forensic psychiatric assessments. The male sex was predominant (94,1%). The mean age was 19,2 years. The perpetrators were unmarried (100%), with a primary school level or less (55,9%), and low socioeconomic level in all cases. They had personal criminal records in 20,6% One-fifth had experienced emotional deprivation in childhood. The father was described as violent in 20,6% of cases. The most common diagnosis were antisocial personality disorder (55,9%) and mental retardation (29,4%). The main offences were homicide and attempted homicide (47.1%), assault and battery (26,5%) and sexual offences (20,6%). The experts had concluded to a “non-criminal responsibility” in 38,2% of cases.

**Conclusions:**

The knowledge of epidemiology of violence perpetrated by adolescents highlights the need for targeted research, policy and programming responses for its prevention.

**Conflict of interest:**

No significant relationships.

